# 

**Published:** 1912-07-20

**Authors:** 


### Buildings Contemplated.

Egypt.—Tenders are invited for the erection of an hospital at Qena, Cairo, and particulars may be had from Sir Aston Webb, C.M.G., Westminster, S.W.

Exminster.—At the Devon County Lunatic Asylum, Exminster, it is proposed to erect a new pathological laboratory from plans by the county architect, Mr. E. H. Harbottle, of Exeter. Tenders are being considered.

Great Yarmouth.—The Guardians have received tenders for increasing infirmary accommodation, the erection of a new block to the Caister Road Female Infirmary, and also for heating apparatus. Consideration of the three tenders is, however, deferred.

Kilmarnock.—A scheme for extending and improving the Kilmarnock Infirmary at an estimated cost of £10,700 is on foot; this sum is exclusive of furnishings.

London.—The Metropolitan Asylums Board has agreed to an expenditure of £900 for providing at the North-Western Hospital a new washing-machine, one 108-inch Decoudun ironing-machine, a new shirt-ironer, a hydro-extractor, and a new engine, together with £325 for building works, including an addition to the ironing accommodation. Similar work at the Western Hospital, estimated to cost £590, was also sanctioned.

Nagyvarad.—The *Zentral-Anzeiger* (Vienna) reports that plans are now prepared for the erection of a public infirmary in Nagyvarad, to contain 460 beds, at an estimated cost of £112,500.

Norwich.—The Committee of Visitors to the Norwich Asylum recommend the Corporation to spend £7,943 on remodelling the premises.

### BUREAU OF INFORMATION.

### Rules for Correspondents.

1. Every letter, on whatever subject, must be accompanied by the coupon to be cut from the back cover (inside page) of The Hospital, current issue, and must contain the name and address of the correspondent with pseudonym for publication if desired.
2. Replies by post cannot be given, save under exceptional circumstances at the Editor's discretion.
3. Letters from Approved Homes in reply to special needs published in the Bureau should state terms and full particulars, and be sent prepaid under cover to the Editor of the Bureau with coupon and name enclosed for identification.
4. Proprietors of homes which have not yet been entered on the List of Approved Homes, but have spare accommodation likely to suit special needs, are invited to write for an application form for registration. The fee for registration is 5s.
5. Special needs of every description relating to those who are sick or in difficulties are made known in these columns free of charge.
6. All communications to be addressed to The Editor of The Hospital, 28 Southampton Street, Strand, London, W.C., and marked "Bureau of Information."

### The Union of Nursing Homes.

### The Object of Maintaining an Approved List.

We invite doctors, nurses, and members of the public to take advantage of the opportunity offered in these columns of making known, free of charge, under initials or pseudonym, the needs of invalids who require accommodation in medical, surgical, or miscellaneous homes. They will be freed from the applications of agents and unsatisfactory homes by our rule of forwarding letters from none but homes whose *bona-fides* have been tested by direct medical and other testimony. A fee of five shillings is charged to Approved Homes after due inquiry, and this covers two announcements of the home in the Bureau: first, on registration; and, secondly, in the half-yearly List. Proprietors of Approved Homes are requested to study the columns of the Bureau every week, and to write personally under cover of the Bureau to any who make known their need for the class of accommodation they have to offer. By this means a strong centre of communication between the public and the *bona-fide* homes is gradually being developed with the aim of meeting applications for accommodation in every part of this and other countries without delay. We do not forward particulars of cases direct to proprietors of homes on the List; we announce them as they are sent in to us in the section of the Bureau reserved for this purpose.

### Training and Employment.

### Massage and Electricity.

Masseuse.—The only satisfactory way of receiving a thorough training in "electricity to be used for patients, under medical supervision," is in the wards of a hospital. Theoretical training is of little value, because, however good the lecturer may be, his teaching must be barren unless he can bring you into actual contact with patients under treatment. Write for particulars of their courses to the West End Hospital, 73 Welbeck Street, London, W., and to the National Hospital, Queen Square, Bloomsbury, W.C. Both these hospitals have excellent schools of massage and electrical treatment open to outside students.

### Secretarial Post.

Sister Dora.—Will you tell your friend to write fully, stating her qualifications and the work she is prepared to do, to G. W. H., under cover of the Bureau, and we will forward it? If she has a genuine interest in the relief of suffering this may prove an opening for her. As she is well educated and a good linguist, business-like, and an expert typist, she is well qualified for secretarial work.

### Empire Nursing.

### Prospects in New Zealand.

Josephine B.—It is a pity that you and your two friends who want to go out to New Zealand have not taken the C.M.B. certificate, though fully trained in other respects. Certified midwives are more needed, especially for the country regions known as "back blocks," than medical and surgical nurses, of whom the Colony trains a very sufficient supply for itself. You will be in a better position on landing if you pay your own passage than if you go out as attendant to some one on the voyage, as these engagements are rarely entered into without an agreement to stay on for a year or two afterwards. On arrival it will be necessary to get registered. We recommend you to write to the Lady Superintendent, The Nurses' Hostel, Wellington, N.Z., and ask what prospect of private work you would find. In any case this would be a good place to stay at on arrival while you are making inquiries. There is undoubtedly an element of risk in going out, and you must be prepared for unfamiliar conditions and an uphill struggle.

### Inexpensive Homes Offered.

### In Nursing Home on N.E. Coast.

Sister A. (103x).—We are very pleased to make it known that you can receive a convalescent or chronic case at one guinea a week in your comfortable Nursing Home near the sea on Durham coast. There are many who would be glad to know of this opportunity. Prepaid replies forwarded.

### For Tired Nurses at Worthing.

Lady Superintendent (104x).—Thanks for kind letter. The offer of a home for tired nurses and others at the low terms of 10s. 6d. a week is very opportune, and may put a holiday within reach of some who would not get a rest at all without such generous help. Prepaid replies forwarded.

### Needs and Helpers.

V. M.—Write for particulars to the Aged Pilgrim's Friend Society, 83 Finsbury Pavement, London, E.C. For a small annual subscription annuities are given and in some cases homes provided. Write also to the Misses Harrison, Secretaries of Homes for the Aged Poor, 86 Penge Road, Anerley, S.W. It would relieve the strain very much if you could secure your friend a free lodging through the latter society. She will have to enter her name and wait her turn in both cases. She will need her birth certificate and should procure it at once. If you will send us on the name and address, we may be able to procure temporary help through one of our Chiswick correspondents. She is evidently past work.

### Letter-Box.

N. T., Boscombe.—We have forwarded your letter to the Home of Rest for Gentlewomen, who will send you particulars. Yes; coupon must be enclosed with every communication sent to us, whether it be a query or merely a letter to be forwarded.

G. W. H.—See reply to Sister Dora. "Zealous One" is not now needing a post.

**The leaflet containing particulars of the Bureau is now ready and will be forwarded free on application.**

### PRESENTATION FOR LONG SERVICE AT HULL.

An interesting presentation was made by Sir James Reckitt, Bart., on behalf of the Hull Royal Infirmary Board, to Mr. William R. Birkwood, whose period of service as barber at this institution all but covered half a century. The recipient expressed some astonishment at this spontaneous gift, and, in thanking the board for their kind cheque, assured the gentlemen present that, though eighty years of age, he still retained a steady hold on that instrument which plays a minor part in the preparatory stage of certain hospital operations.

